# Hazardous Doping for Photo-Electrochemical Conversion: The Case of Nb-Doped Fe_2_O_3_ from First Principles

**DOI:** 10.3390/molecules201119668

**Published:** 2015-11-04

**Authors:** Natav Yatom, Maytal Caspary Toroker

**Affiliations:** Department of Materials Science and Engineering, Technion—Israel Institute of Technology, Haifa 32000, Israel; nyatom@tx.technion.ac.il

**Keywords:** water splitting, Density Functional Theory, DFT+U, iron oxides, doping

## Abstract

The challenge of improving the efficiency of photo-electrochemical devices is often addressed through doping. However, this strategy could harm performance. Specifically, as demonstrated in a recent experiment, doping one of the most widely used materials for water splitting, iron(III) oxide (Fe_2_O_3_), with niobium (Nb) can still result in limited efficiency. In order to better understand the hazardous effect of doping, we use Density Functional Theory (DFT)+U for the case of Nb-doped Fe_2_O_3_. We find a direct correlation between the charge of the dopant, the charge on the surface of the Fe_2_O_3_ material, and the overpotential required for water oxidation reaction. We believe that this work contributes to advancing our understanding of how to select effective dopants for materials.

## 1. Introduction

Improving the efficiency of solar energy conversion in a photo-electrochemical device is challenging. Achieving the goal of enhancing efficiency relies on at least two material properties: one is having high charge conductivity and the second is having good catalytic ability [1]. Unfortunately, one of the commonly used materials for water splitting, iron(III) oxide (Fe_2_O_3_), lacks both these properties [2].

Successful advances have been demonstrated with point defects such as doping [3,4,5,6,7,8]. N-type doping of Fe_2_O_3_ with elements such as Ti or Si increases the number of electron charge carriers and therefore can boost electron conductivity [9,10]. Furthermore, Si has an advantage over Ti since the latter creates low energy states that can “trap” electron carriers [11]. Therefore, selecting dopants carefully is crucial for improving electron conductivity.

Despite improvements in electronic conductivity, recent studies show that doping can harm catalysis. For example, doping Fe_2_O_3_ with Ti, Si, or Pt can unfavorably increase the overpotential for water oxidation [12,13,14]. The trends in changing the overpotential upon doping was related to the adsorbing energies of the reaction intermediates. Among several dopants that were considered, the most effective dopants for favorably reducing the overpotential were discovered to be Ni and Co [12].

In this paper, we use Density Functional Theory (DFT)+U in order to demonstrate how n-type doping could be hazardous for catalysis. This is the first study where we calculate the free energies involved in water oxidation of Nb-doped Fe_2_O_3_. We select the Nb dopant for two reasons. First, recent experiments show that doping Fe_2_O_3_ with Nb unfavorably increases overpotential [15]. Second, Nb has a formal charge of +5 which is lower than the charge of +4 in conventional n-type dopants of Fe_2_O_3_. We find that Nb overcharges the surface of Fe_2_O_3_ and that this has a direct consequence on catalysis.

## 2. Computational Details

The VASP simulation package (5.3 version, VASP group at University of Vienna, Vienna, Austria) was used for all spin-polarized Density Functional Theory (DFT) calculations [16,17] with the PBE (Perdew, Burke, and Ernzerhof) [18] exchange-correlation (XC) functional. We chose the Dudarev *et al.* DFT+U formalism [19] because it better describes the strong correlated electrons of first row transition metals [20,21,22,23]. An *ab-initio* derived value of U − J = 4.3 eV [24] was chosen for Fe atoms, while zero for O, Nb and H atoms that are closed-shell in their corresponding stable oxidation states. Nuclei and core electrons were described by PAW potentials [25,26] such that only the 6, 14, 11 and 1 valence electrons of O-2s^2^2p^4^, Fe-3p^6^3d^6^4s^2^, Nb-4p^6^5s^1^4d^4^, and H-1s^1^, respectively, were considered explicitly.

Several computational settings were used to achieve convergence. Ion positions were relaxed using conjugated gradient method and the stopping criterion for the ionic relaxation was chosen for all forces to be smaller than 3 × 10^−2^ eV/Å. No symmetry was imposed. An energy cutoff of 700 eV converged the total energy to less than 1 meV per atom. 4 × 4 × 4 and 3 × 3 × 1 gamma-centered k-space grid for the bulk and surface, respectively converged the total energy to 1 meV. Ionic relaxation was done with Gaussian smearing method with smearing width of 0.01 eV for fast relaxation, and the last iteration was done with Tetrahedron method with Blöchl corrections [27,28].

The primitive unit cell of Fe_2_O_3_ is rhombohedral and contains two Fe_2_O_3_ stoichiometric units. Bulk unit cells were cleaved at the (0001) plane which is one of stable surfaces of Fe_2_O_3_ [29,30]. Due to similarity of ionic radii [31], the Nb was inserted in the slab as an Fe substitution defect and not as interstitial defect as detected experimentally [32]. Although Nb has some solubility in Fe_2_O_3_ of up to 2 ± 1 at% [33], we chose to substitute Nb in a slab with 32 atoms (7 at%) in order to have a direct comparison to pervious calculations with other dopants using the same level of theory (DFT+U) [12]. Furthermore, when we use a longer slab with 39 atoms (5 at%) the overpotential changes by less than 0.02 eV. In addition, previous calculations show little dependence of doping concentration on the calculated free energies [12]. The initial position of terminating hydrogen atoms was placed according to the information given in previous calculations [12]. We do not include additional water molecules above the slab since previous work showed that the first water monolayer has little influence on the calculated free energies [12]. All slab calculations were converged with a 10 [Å] vacuum.

The analysis is based on the conventional approach developed by Nørskov for modeling surface chemistry. The model is based on having a series of surface slab models representing intermediate reactions. The free energy required for each reaction step is calculated by solving the Konh-Sham equations self-consistently for each reaction intermediate. We consider the following mechanism for water oxidation, as previously suggested [34]:

(1) $H_{2}O+*\to *OH_{2}$
(2) $*OH_{2}\to *OH+H^{+}+e^{-}$
(3) $*OH\to *O+H^{+}+e^{-}$
(4) $H_{2}O+*O\to *OOH+H^{+}+e^{-}$
(5) $*OOH\to O_{2}+*+H^{+}+e^{-}$

where * denotes oxygen vacancy site on the surface and *OH_2_, *OH, *O, and *OOH are adsorbates. The corresponding free energies were calculated by subtracting the total energies of reactants and products (for example, ∆*G*_1_ is calculated for reaction 1. We added the reported zero point energy (ZPE) corrections and entropic contributions of pure Fe_2_O_3_ since composition was shown to have a negligible effect on the results [12]. The overpotential (O.P) is defined as voltage needed to add to the calculated electrochemical potential of:

(1) $$\frac{\sum_{i=1}^{5}\Delta G_{i}}{4e}=\frac{\Delta G_{\left({\mathrm{2H}}_{2}O_{\left(l\right)}\to 2H_{2\left(g\right)}+O_{2\left(g\right)}\right)}}{4e}=\frac{4.43\left[eV\right]}{4e}=1.11\left[V\right]$$

in order to obtain the reaction potential:

(2) $$\Phi_{rx}=\frac{\max{\left\{\Delta G_{i}\right\}}_{i=1}^{5}}{e}$$

in which all reaction steps free energies are negative, such that the reaction is thermodynamic spontaneous:

(3) $$\text{O.P}=\Phi_{rx}-1.11\left[V\right]$$

## 3. Results and Discussion

This section contains our free energy calculations for water oxidation on Nb-doped Fe_2_O_3_(0001) and a comparison to previously-studied undoped Fe_2_O_3_ and Ni- and Pt-doped Fe_2_O_3_. We find that the overpotential upon Nb doping is largest, according to the following relation: Nb > Pt > Fe > Ni, which we correlate to the oxidation states Nb^+5^ > Pt^+4^ > Fe^+3^ > Ni^+2^, as detailed below.

**Figure 1:**
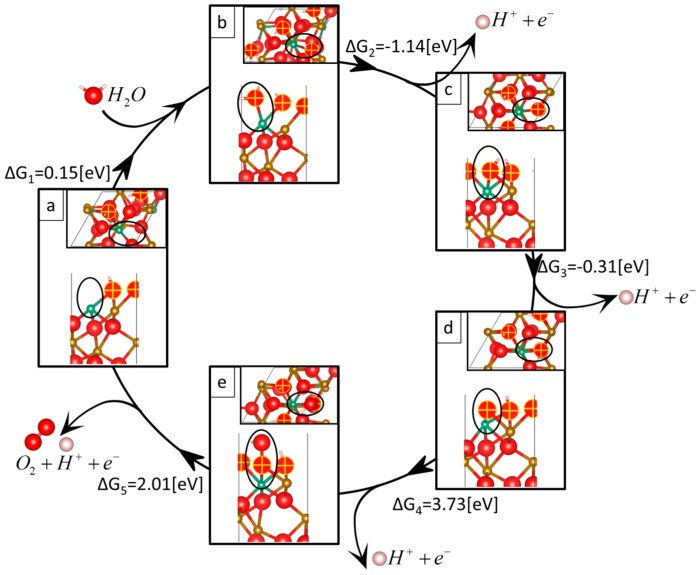
Catalytic cycle of the water oxidation reaction on Nb-doped Fe_2_O_3_(0001). Red, gold, green, and white spheres represent O, Fe, Nb, and H atoms, respectively. The insets show a top view of each reaction intermediate slab structure. The active site is circled in black. Created with VESTA [35].

Calculating the free energies for Nb-doped Fe_2_O_3_ surface reveals that the first chemical bond breaking is favorable (as seen in Figure 1, ∆*G*_2_ = −1.14 eV). This first dehydrogenation is thermodynamically easy since reaction 2 involves extracting an electron from a surface that contains excess Nb-donated electrons. The negative free energy at the first dehydrogenation is compensated by a large free energy at later reaction steps. Subsequent reaction intermediates have less electron charge and therefore are more stable and have a corresponding larger free energy. As a result, the overpotential significantly increases upon Nb doping.

That is, the amount of charge generated by the dopant at the surface affects the overpotential. Since Nb generates two electrons, the free energies involved in taking away these excess electrons are negative (as seen in Figure 1, reactions 2 and 3). The number of electrons generated depends on the atom type and since the surface prefers to remain neutrally charged then this also affects the overpotential.

**Figure 2:**
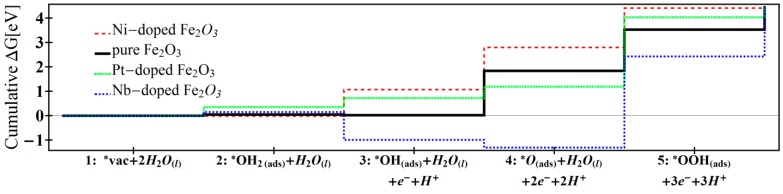
Cumulative free energies for water oxidation on Ni-doped, pure, Pt-doped, Nb-doped Fe_2_O_3_. Values for non-Nb doped materials are obtained from refs [12,13].

The overpotential for water oxidation on Fe_2_O_3_ depends on the dopant atom type. As seen in Table 1 and Figure 2, the overpotential can be ranked according to the following relation: Nb > Pt > Fe > Ni, where Nb doping generates the highest overpotential.

The high overpotential required to induce water oxidation in Nb-doped Fe_2_O_3_ results from the surface charging brought by the dopant. For example, in contrast to Nb doping, Ni does not donate excess electrons and the first dehydrogenation in Ni-doped Fe_2_O_3_ requires a positive free energy (∆*G*_2_ = 1.08 eV). Free energy is required to extract a proton and an electron from the water-adsorbed surface that prefers to stay neutrally charged. The rest of the free energy needed to oxidize water is distributed over three dehydrogenation reactions (as seen in Table 1: reactions 2–4). As a whole, doping Fe_2_O_3_ with Ni results in the best and lowest overpotential.

**Table 1:** Free energies of intermediate water oxidation reactions for Ni-doped, pure, Pt-doped, and Nb-doped Fe_2_O_3_. Units are in eV.

	Reaction 1	Reaction 2	Reaction 3	Reaction 4	Reaction 5	Overpotential
**Ni-doped [12]**	−0.01	1.08	1.73	1.61	0.03	0.62
**Pure [12]**	0.05	−0.03	1.82	1.68	0.91	0.71
**Pt-doped [13]**	0.35	0.36	0.47	2.83	0.40	1.72
**Nb-doped**	0.15	−1.14	−0.31	3.73	2.01	2.62

**Figure 3:**
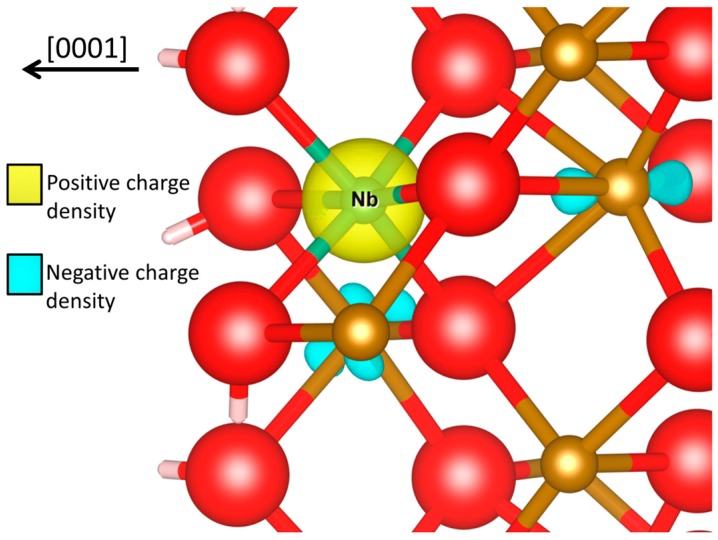
Charge density difference plot for the *OH intermediate of Nb-doped *vs.* pure Fe_2_O_3_. Calculated by subtracting the electron density of the pure Fe_2_O_3_ from the Nb-doped Fe_2_O_3_*OH intermediate at the fixed ionic positions of the latter. Red, gold, green and white spheres denote O, Fe, Nb and H atoms, respectively. Negative and positive charge density iso-surfaces (±0.07 [e/Bohr^3^]) are in blue and yellow, respectively, where negative being absence of electrons. Created with VESTA visualizing software [35].

Therefore, the charge of the dopant has a critical role. The Nb dopant is n-type in Fe_2_O_3_ and has a corresponding oxidation state of +5 (according to a Müllikan magnetic moment of zero). This can be visualized in Figure 3, where Nb donates two electrons that are located on two iron atoms. 

Therefore, Nb donates twice as much charge compared to the Pt dopant which we have recently studied and has an oxidation state of +4 [13]. In contrast, previous calculations show that Ni is a p-type dopant and has an oxidation state of +2 [12]. Therefore, the relation between the oxidation states is: Nb^+5^ > Pt^+4^ > Fe^+3^ > Ni^+^^2^, where Nb charges the Fe_2_O_3_ surface the most.

**Figure 4:**
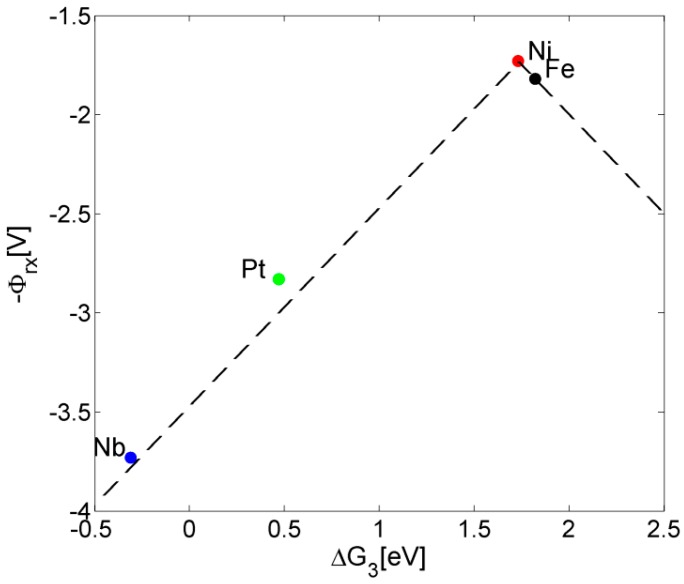
Volcano plot for Fe_2_O_3_ showing location of Nb-doping at the far end of the curve.

Finally, the results can be positioned on a Volcano curve that correlates between the reaction potential Φ*_rx_* and the surface binding energy of adsorbates [12,34,36,37,38]. As can be seen in Figure 4, the reaction potential Φ*_rx_* for Nb is located on the Volcano curve that was previously obtained for other dopants of Fe_2_O_3_ (dashed line in Figure 4) [12], and therefore maintains the known scaling relationship between overpotential and reactants adsorption energies. Nb is positioned at the lower part of the curve with an outstandingly high reaction potential Φ*_rx_* (Figure 4) and overpontential (Equation (3)).

## 4. Conclusions

We modeled water oxidation on Nb-doped Fe_2_O_3_(0001) and found that the overpotential is significantly higher than for pure Fe_2_O_3_ or for Fe_2_O_3_ doped with elements that have a smaller oxidation state than Nb^+5^. We explained this hazardous effect of increasing the overpotential in terms of surface charging: overcharging the surface at the beginning of the reaction via Nb-doping causes the first as well as the second dehydrogenation to be thermodynamically spontaneous (as we can see in Table 1, reactions 2 and 3 have a negative ∆G for the Nb-doped case). Overall, the free energies are not distributed evenly throughout the reaction intermediates and therefore the overpotential is extremely large. By comparing to other dopants studied previously in the literature, we find a direct correlation between the reaction overpotential and the oxidation state of the dopant. Our results agree with experiment that show an increase in overpotential upon Nb-doping [15]. However, we note that previous experiments show that water splitting efficiency improves upon doping with Nb [15,39,40], probably since other factors, such as band gap, band edge positions, and electronic conductivity affect the overall photon-to-hydrogen conversion efficiency; we shall address other effects of Nb in our next publication. We envision that our new understanding of the effect of doping on water oxidation will be useful for selecting effective dopants for surface catalysis.

## Supplementary Material

Unit cell geometries are provided. Supplementary materials can be accessed at: http://www.mdpi.com/1420-3049/20/11/19668/s1.
