# Supplementary material for: Hazardous Doping for Photo-Electrochemical Conversion: The Case of Nb-Doped Fe2O3 from First Principles
Source: Molecules. 2015 Nov 4;20(11):19900–6. doi: 10.3390/molecules201119668 (PMC6332189; doi:10.3390/molecules201119668)
Supplement: Supplementary file 1 [file molecules-20-19668-s001.pdf]

molecules

MDPI

Supplementary Informations

The relaxed geometries of *, *OH_2, *OH, *O, *OOH intermediates slabs are provided below according to the following order: title, lattice vectors in Cartesian coordinates, atom types and numbers, respectively, and atom locations in fractional coordinates with respect to the lattice vectors. The atom coordinates are given according to the ordering in the atom type row (format of VASP).

The corresponding slabs illustrations are in Figure S1.

(1) *Vacancy

Nb doped Fe_2O_3 (0001) slab with one active site in * state and two *OH sites.

```
   1.00000000000000
     5.0977578162999997    0.0000000000000000    0.0000000000000000
     2.5488789081999998    4.4147877713000003    0.0000000000000000
     0.0000000000000000    0.0000000000000000   24.2761898040999995
   O     Fe    H     Nb
    13     6     4     2
Direct
  0.6214553488349210  0.9835458122313483  0.7080738225701424
  0.2839400714647411  0.6348787228585664  0.7057864818628303
  0.9706418296912247  0.0159933633834086  0.6131558180805001
  0.6210234410950690  0.6328815246763710  0.6047723719115154
  0.3228909514340685  0.3533829257868106  0.6001033860569009
  0.9450635983163593  0.3559855739132445  0.5017559001712826
  0.2569299128935114  0.7254122990718130  0.4964091007506610
  0.5739624030412216  0.0482612786191723  0.5043598534454574
  0.2803256989020184  0.2997378923691159  0.3805999983086252
  0.9496095814373149  0.9718965293577781  0.3982143731993872
  0.6809115136274499  0.6715819508045584  0.4024535047964335
  0.3701618849977564  0.5705964282500844  0.2925692672737128
  0.7035594197111408  0.8504366148744253  0.2822457467466677
  0.9277665781661426  0.6635468054457121  0.6480902056797433
  0.9042537030868445  0.7171364629540520  0.4689691972066399
  0.2833401203179591  0.0239735465480351  0.4399902636206789
  0.2822148622716867  0.0102868333237254  0.5604491556153448
  0.5838874849319353  0.3989137488998580  0.5341238624582942
  0.6336889864447812  0.3718669395283101  0.3622692091030260
  0.4651022103331499  0.9267075534772857  0.7132908025387579
  0.5499783666586140  0.7882327528127391  0.2761394481717474
  0.2529518540395586  0.5791377557552906  0.7425251189896045
  0.4243658394429062  0.4098798073410350  0.2653870942459378
  0.6259874621130592  0.2952084150414365  0.6544559512814914
  0.9846447587465903  0.6664633486758049  0.3443930299146345
```

(2) *OH_2

Nb doped Fe_2O_3 (0001) slab with one active site in *OH_2 state and two *OH sites.

```
     5.0977578162999997    0.0000000000000000    0.0000000000000000
     2.5488789081999998    4.4147877713000003    0.0000000000000000
     0.0000000000000000    0.0000000000000000   24.2761898040999995
   O     Fe    H     Nb
    15     6     8     2
```

Direct

0.6622763840815580	0.9723061874065451	0.7037834001548759
0.0017348178103944	0.2136629400615959	0.7267536681270599
0.3169657229739258	0.6334178020438372	0.7130923664434761
0.9509752150471726	0.0396514672953145	0.6069986145524027
0.5913514950214420	0.6645514211362809	0.6021084866475590
0.3194281284898040	0.3563346264271772	0.6038307504268303
0.9241168033770464	0.3799332781449678	0.4991591885267681
0.2385763452631195	0.7416948362702911	0.4966638956723273
0.5583888089741578	0.0681534585428878	0.5023942497881464
0.2705982703997591	0.3314263538685225	0.3874190688922283
0.9479764591112527	0.9641773130286779	0.3981104125022270
0.6445518275525544	0.6873620616544613	0.3961239615037471
0.3220486887732790	0.6933428376977204	0.2961418641814788
0.7127138716371917	0.0289047317464721	0.2895187829019221
0.0758944479843677	0.2689126396760457	0.2757084355779824
0.9285401178291295	0.6709435146249945	0.6382151022860398
0.8875431367036200	0.7383280026906007	0.4659877357303301
0.2694195032160707	0.0284777010759214	0.4403071120782727
0.2681685821631987	0.0384516942116733	0.5577396472192078
0.5689147398012722	0.4154900334379050	0.5335581876514652
0.6331264924545223	0.3613684657209717	0.3566347986337206
0.4894441671418264	0.9444439233343047	0.7095686569244677
0.5216325564256081	0.0751106305584983	0.2725126957682420
0.2378280047356327	0.5346300742726413	0.7368449919189075
0.3349949284941047	0.6117047915382159	0.2593220943936387
0.8648613219220209	0.2667959714898203	0.7581061251279166
0.8921551819368716	0.2710915814246917	0.2622349332875302
0.1148509919126468	0.9784861957026578	0.7247715109486705
0.1887619718117790	0.0449341139188988	0.2889344889692822
0.6454620914536164	0.3233532860214936	0.6542615446260669
0.9737598665010552	0.6525569749759157	0.3446173915372199

(3) *OH

Nb doped Fe_2O_3 (0001) slab with three active site in *OH state.

5.0977578162999997	0.0000000000000000	0.0000000000000000
2.5488789081999998	4.4147877713000003	0.0000000000000000
0.0000000000000000	0.0000000000000000	24.2761898040999995

O	Fe	H	Nb
15	6	6	2

Selective Dynamics

Direct

0.6240143236728102	0.0022596420943373	0.7024177426292439
0.9400827449792573	0.3427665996994023	0.7038352272085717
0.3070318855673193	0.6469032121514644	0.6981468348874387
0.9855794299910201	0.0028058266245523	0.6018798746284730
0.6386051828433565	0.6378759006130394	0.6037473945852128
0.3320825333412856	0.3082275073220018	0.6039669317898273
0.0335302939996112	0.2836320390144849	0.5005583996614362

0.3286247607159893	0.6934529189965204	0.4994983880632518
0.6230178931924684	0.9885512036590711	0.5000668321384367
0.2886650016655139	0.3389464916245331	0.3963099056612762
0.0003869536906080	0.9742240270660929	0.3982021144725891
0.6525119898030596	0.6687879787040529	0.3961619480880216
0.2950403865520315	0.6343464723176311	0.2962656852544328
0.6384281131814049	0.9746758239924844	0.2976759100056583
0.9661023125732058	0.3301080574755275	0.3019375739484005
0.9621940925516768	0.6675865594168329	0.6492173133902313
0.9942170152304683	0.6550536466783328	0.4663828285738632
0.3211020312621358	0.9952982516610405	0.4429756009276247
0.3272901858376898	0.9818713246230217	0.5571106544559612
0.6607609008010513	0.3219269051024867	0.5336995487659237
0.6419543708811943	0.3094279220155585	0.3508603853333199
0.4400971054458793	0.9847494554627474	0.7034561754772355
0.4370157138917747	0.9921687181251926	0.2966434214211091
0.2871181589110208	0.5731580732393269	0.7339602557118567
0.3086578812401015	0.5127648762544439	0.2643381330776293
0.8320777416070797	0.4644444352818979	0.7357503959617304
0.8725714729501561	0.4037177959209828	0.2661107361027959
0.6564759130145461	0.2931388104311878	0.6478858590706764
0.9618205926062728	0.6837984374317543	0.3522059887078441

(4) *O

Nb doped Fe_2O_3 (0001) slab with one active site in *O state and two *OH sites.

5.0977578162999997	0.0000000000000000	0.0000000000000000
2.5488789081999998	4.4147877713000003	0.0000000000000000
0.0000000000000000	0.0000000000000000	24.2761898040999995

O	Fe	H	Nb
15	6	4	2

Direct

0.6266189867760374	0.9786178159108871	0.6987820832818699
0.9661929906906934	0.3180985042819628	0.6952045027105740
0.2915250004683401	0.6396716346184661	0.6931885308956112
0.9387584421380453	0.0161880999527426	0.5950542658935021
0.6173333859790944	0.6231027441359984	0.6000951378180233
0.3371902775598485	0.3040414760141843	0.5957915382942218
0.9843650267851345	0.2926087854646937	0.5003305843873207
0.2936150688651011	0.6790879295080714	0.4998305864237054
0.5985150824776895	0.9833373748304979	0.4998847069318941
0.2504605425190860	0.3458373013432379	0.4041396673567519
0.9732622168746303	0.9423953581501081	0.4030205483917300
0.6502744806310474	0.6608532249846562	0.4020616181066146
0.3092670013946659	0.6499989811659077	0.2987037177155329
0.6264268425622461	0.9984981616665110	0.3078427154098691

0.9482878959835048	0.3339175057572845	0.3067022259779080
0.9663483773023813	0.6504515994364795	0.6383588096116668
0.9568442300276701	0.6524331039905817	0.4646761735227827
0.2907802099061385	0.9795467181570174	0.4404628982333989
0.3021815656545925	0.9850375176875943	0.5596917155211258
0.6214666992568141	0.3202095919963810	0.5351310178003033
0.6219185475383640	0.3238767469849577	0.3636399343367032
0.4403410410184350	0.9651814429702625	0.6997935603397849
0.4419738137966291	0.9843093479446665	0.3031122650311318
0.2369581936799747	0.6001176542258762	0.7294800084530877
0.4035347463492656	0.5283189154279100	0.2659634850889248
0.6787501601881800	0.2845987140458135	0.6579660912665162
0.9838711835763689	0.6263318103472741	0.3410936681994343

(5) *OOH

Nb doped Fe_2O_3 (0001) slab with one active site in *OOH state and two *OH sites.

5.0977582932000001	0.0000000000000000	0.0000000000000000
2.5488791466000000	4.4147881841999999	0.0000000000000000
0.0000000000000000	0.0000000000000000	24.2761898040999995

O	Fe	H	Nb
17	6	6	2

Direct

0.6458251427934432	0.0344687813385619	0.7089750811973080
0.9630793825033304	0.3377401123596460	0.7017427080867424
0.3041716665083785	0.6711478727301823	0.7002581630576742
0.9835992105642712	0.0285723349968683	0.6026990466199966
0.6482561779171548	0.6575607986343114	0.6084911650501797
0.3141918593465551	0.3432427707144921	0.6043358799361335
0.1251084368075421	-0.1605465756825322	0.3761968373412518
0.3431882193365164	0.6466413314092947	0.4960888624126415
0.6273562640619403	0.9578344852624684	0.4866141351550065
0.2806327646779987	0.3123312155814658	0.3954707502133011
0.9970318944248683	0.9474355770600232	0.3959005036163764
0.6484531601971409	0.6342253881775455	0.3931437868970341
0.2831875504553165	0.6062953790330705	0.2945929992350459
0.6324550580953602	0.9370307047784892	0.2955448074264904
0.9704978176536199	0.2971801958472934	0.3001055346315532
0.9205542087770118	0.3329872812850978	0.7614226528026515
0.9128415897816781	0.3782146370821289	0.2414378474222562
0.9558707535003044	0.6961689683633050	0.6545258180667788
0.0141334744683973	0.6366218635839576	0.4614199862379255
0.3317264103086682	0.9387814320345077	0.4420850849362108
0.6333024656444850	0.0569250998787040	0.5557782233410076
0.8885933415663305	0.7819381607223661	0.6630052998167419
0.6353225766065114	0.2796128771491305	0.3468393224324192

0.4668677716252214	0.0127988406301015	0.7157429583370547
0.4241923818141699	0.9694630321740983	0.2959814885802750
0.2918974911813166	0.6045503972936483	0.7373899953962301
0.2861705427115045	0.5110826466518124	0.2594479565438408
0.8412440522090265	0.1909571238063253	0.7624215008650146
0.7761528144194907	0.6008225112209593	0.2443765014693818
0.6381850298284730	0.3410098551947973	0.6508588888734275
0.9615701748244896	0.6505986740261894	0.3505862566257061

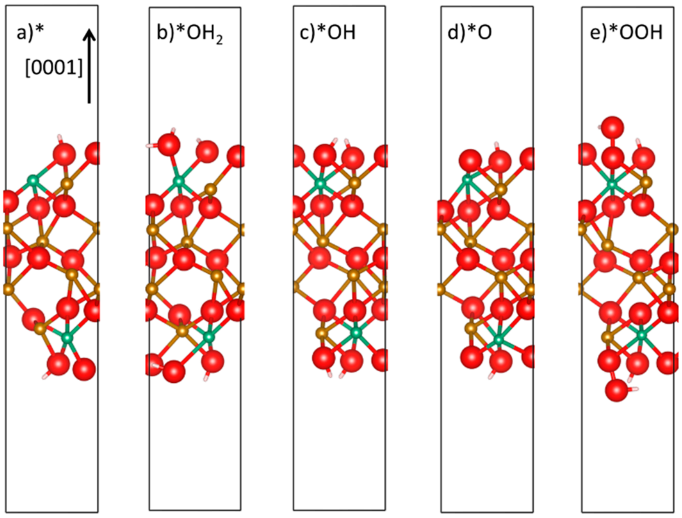

Figure S1. Slabs for *, *OH_2, *OH, *O, *OOH intermediates are illustrated in a, b, c, d, and e, respectively. Red, gold, green, and white spheres represent O, Fe, Nb, and H atoms, respectively. The black arrow indicates the [0001] crystal direction normal to the hematite (0001) surface. The black frame is the cell's boundary. Created with VESTA: Momma, K.; Izumi, F. VESTA: A three-dimensional visualization system for electronic and structural analysis. *J. Appl. Crystallogr.* 2008, *41*, 653–658.

© 2015 by the authors; licensee MDPI, Basel, Switzerland. This article is an open access article distributed under the terms and conditions of the Creative Commons by Attribution (CC-BY) license (http://creativecommons.org/licenses/by/4.0/).
